# Conservative versus flexible physiological and metabolic acclimation of Norway spruce and European beech under elevated CO_2_, nitrogen supply and water availability

**DOI:** 10.1093/treephys/tpag125

**Published:** 2026-09-08

**Authors:** Kojo Kwakye Ofori-Amanfo, Karel Klem, Petr Holub, Barbora Veselá, Michal Oravec, Kristýna Večeřová, Thomas Agyei, Roman Sándor, Stanislav Juráň, John Grace, Michal V Marek, Otmar Urban

**Affiliations:** Global Change Research Institute, Czech Academy of Sciences, Bělidla 986/4a, Brno 603 00, Czech Republic; Global Change Research Institute, Czech Academy of Sciences, Bělidla 986/4a, Brno 603 00, Czech Republic; Department of Agrosystems and Bioclimatology, Faculty of AgriSciences, Mendel University in Brno, Zemědělská 1665/1, Brno 613 00, Czech Republic; Global Change Research Institute, Czech Academy of Sciences, Bělidla 986/4a, Brno 603 00, Czech Republic; Global Change Research Institute, Czech Academy of Sciences, Bělidla 986/4a, Brno 603 00, Czech Republic; Global Change Research Institute, Czech Academy of Sciences, Bělidla 986/4a, Brno 603 00, Czech Republic; Global Change Research Institute, Czech Academy of Sciences, Bělidla 986/4a, Brno 603 00, Czech Republic; Global Change Research Institute, Czech Academy of Sciences, Bělidla 986/4a, Brno 603 00, Czech Republic; Department of Biological Sciences, University of Energy and Natural Resources, Sunyani–Berekum Road (Fiapre), Sunyani Municipal District, Post Office Box 214, Sunyani BS-0061-2164, Ghana; Earth Observation, Research and Innovation Center, University of Energy and Natural Resources, Sunyani–Berekum Road (Fiapre), Sunyani Municipal District, Post Office Box 214, Sunyani BS-0061-2164, Ghana; Global Change Research Institute, Czech Academy of Sciences, Bělidla 986/4a, Brno 603 00, Czech Republic; Global Change Research Institute, Czech Academy of Sciences, Bělidla 986/4a, Brno 603 00, Czech Republic; Global Change Research Institute, Czech Academy of Sciences, Bělidla 986/4a, Brno 603 00, Czech Republic; School of GeoSciences, University of Edinburgh, Crew Bldg, Kings Bldgs, Alexander Crum Brown Rd, Edinburgh EH9 3FF, UK; Global Change Research Institute, Czech Academy of Sciences, Bělidla 986/4a, Brno 603 00, Czech Republic; Department of Biological Sciences, University of Energy and Natural Resources, Sunyani–Berekum Road (Fiapre), Sunyani Municipal District, Post Office Box 214, Sunyani BS-0061-2164, Ghana; Institute of Management, Slovak University of Technology, Vazovova 5, Bratislava 812 43, Slovakia; Global Change Research Institute, Czech Academy of Sciences, Bělidla 986/4a, Brno 603 00, Czech Republic

**Keywords:** abscisic acid (ABA), amino acid metabolism, photosynthetic downregulation, phenolics, phenylpropanoid pathway, stomatal regulation, water-use efficiency

## Abstract

Elevated atmospheric carbon dioxide (CO_2_) (EC) can enhance photosynthesis, biomass accumulation and water-use efficiency (WUE), but responses depend on water and nutrient availability and differ among tree species. Multifactorial studies addressing these interactions remain scarce. We examined interactive effects of EC (~700 μmol mol^−1^), nitrogen supply (nitrogen addition, N^+^; no nitrogen addition, N^−^) and water availability (well-watered, WW; drought-stressed, DS) on physiological and metabolic responses of more anisohydric European beech (*Fagus sylvatica* L.), which maintains stomatal opening longer during drought, and more isohydric Norway spruce (*Picea abies* (L.) Karst.), which closes stomata earlier to stabilize leaf water status. Across both species, EC produced the largest shifts in gas exchange and metabolite profiles, while water and nitrogen availability strongly modified these responses. Elevated CO_2_ generally increased net CO_2_ assimilation and WUE and reduced stomatal conductance, although responses differed between species and treatments; WUE was highest under EC × DS × N^+^. Species divergence was most evident in biochemical acclimation. In beech, EC markedly reduced photosynthetic capacity, reflected in lower Rubisco carboxylation capacity (*V*_Cmax_) and electron transport rate (*J*_max_) across water and nitrogen treatments. This response was accompanied by lower succinate and foliar nitrogen and by resource-dependent shifts in amino acids, phenolic and stress-related metabolites, consistent with greater metabolic reorganization and photosynthetic downregulation. In contrast, spruce maintained comparatively stable *V*_Cmax_ and *J*_max_ under EC, including under DS and N^−^ conditions, together with comparatively stable primary metabolism and sustained phenolic-based defence. Overall, beech showed greater physiological and metabolic flexibility but stronger downregulation of photosynthetic capacity under EC, whereas spruce maintained a more conservative physiological strategy and greater biochemical stability. These contrasting responses highlight species-specific acclimation to CO_2_ enrichment and its modulation by water and nitrogen availability.

## Graphical Abstract

**Graphical Abstract:**
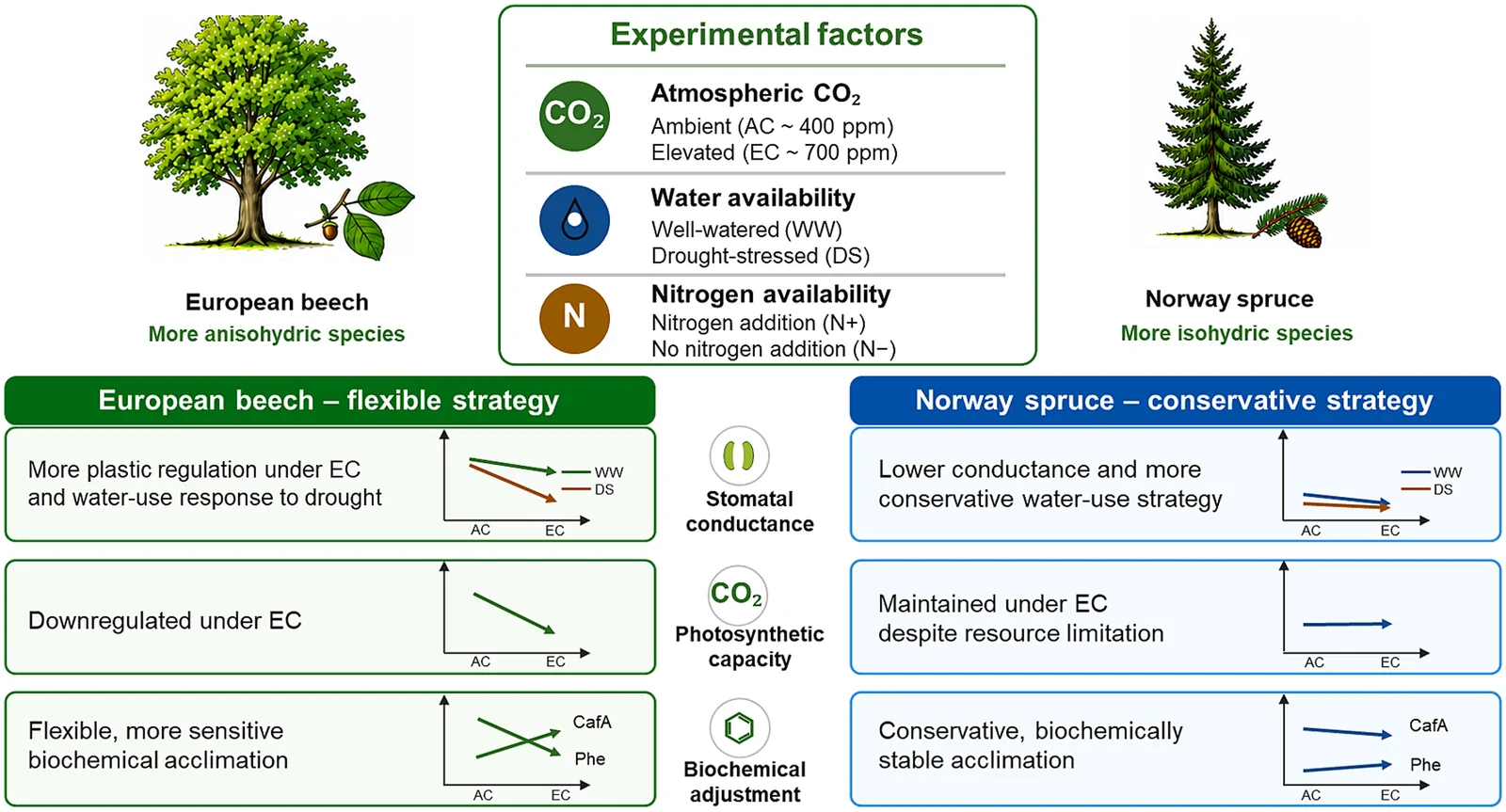

## Introduction

The atmospheric concentration of carbon dioxide (CO_2_) has been steadily rising due to anthropogenic activities such as fossil fuel combustion and land-use change (Sitch et al. 2024). This increase is a major driver of global climate warming (Matthews and Wynes 2022) and is accompanied by altered precipitation and temperature patterns, as well as more frequent and intense droughts and heatwaves (Mukherjee and Mishra 2021). Rising CO_2_ directly affects plant physiology and terrestrial ecosystem functioning (Norby and Luo 2004, Higgins et al. 2023), mainly by stimulating photosynthesis in C_3_ plants (reviewed in Poorter et al. 2022). This stimulation results from increased carboxylation efficiency of ribulose-1,5-bisphosphate carboxylase/oxygenase (Rubisco) relative to its oxygenation activity (Bowes 1991, Jensen 2000). Elevated CO_2_ often reduces stomatal conductance (Medlyn et al. 2001, Ainsworth and Rogers 2007), thereby enhancing water-use efficiency by maintaining high photosynthetic rates while limiting transpirational water loss (Leakey et al. 2009, Poorter et al. 2022).

While short-term exposure to elevated CO_2_ typically enhances photosynthesis, long-term exposure over months or growing seasons can lead to photosynthetic acclimation, often involving downregulation of photosynthetic capacity (Luo et al. 1999, Urban 2003). These responses have frequently been associated with the accumulation of non-structural monosaccharides, particularly hexoses (glucose, fructose, galactose), which may act as signalling molecules (Sheen et al. 1999, Thompson et al. 2017). Sugar-sensing pathways involving hexokinase, Snf1-related protein kinase 1 and trehalose-6-phosphate regulate genes controlling photosynthesis, nutrient uptake and transport and broader metabolic processes (Paul and Pellny 2003, Thompson et al. 2017). For example, doubled atmospheric CO_2_ has been shown to reduce Rubisco content in both Norway spruce and European beech (Košvancová et al. 2009), the two species investigated here. The long-term response of plants to elevated CO_2_ therefore depends on whether photosynthetic capacity can be maintained under concurrent environmental constraints (Körner 2022, Poorter et al. 2022, Ofori-Amanfo et al. 2023). In particular, stomatal regulation, the balance between carbon gain and transpirational water loss, and the maintenance of biochemical capacity for carboxylation and electron transport are likely to determine whether elevated CO_2_ enhances productivity or promotes photosynthetic acclimation. These responses are closely linked to carbon source–sink balance and to water and nitrogen availability, with elevated CO_2_ often increasing leaf C:N stoichiometry (Cotrufo et al. 1998, Urban 2003).

Elevated CO_2_ also modifies metabolism beyond photosynthetic carbon gain. Non-structural saccharides may interact with plant hormones that regulate growth and development (Thompson et al. 2017). For instance, elevated CO_2_ has been linked to increased auxin biosynthesis that promotes root development (Lilley et al. 2012), but also to reduced jasmonic acid synthesis, potentially weakening defence responses. Elevated CO_2_ can also influence secondary metabolism, which is essential for plant defence, stress responses and environmental interactions (Peñuelas and Estiarte 1998). Early studies suggested enhanced accumulation of carbon-rich secondary metabolites derived from the shikimate pathway, such as phenolics (Lindroth et al. 1993, Peñuelas et al. 1997), consistent with the source–sink balance hypothesis (Luo et al. 1999). More recent ecometabolomic studies, however, have revealed heterogeneous responses (Sardans et al. 2020), indicating that these biochemical adjustments are modulated by co-occurring environmental factors. For example, phenolic and lignin accumulation in Norway spruce were unaffected by doubled CO_2_ under cold mountainous conditions (Lhotáková et al. 2012), and terpenoid responses range from reductions (e.g. Peñuelas et al. 1997) to increases in isoprene and monoterpenes (e.g. Himanen et al. 2009). Likewise, although elevated CO_2_ tends to increase plant carbon:nitrogen (C:N) ratios (Cotrufo et al. 1998, Urban 2003), and nitrogen-based secondary metabolites were therefore expected to decline (Peñuelas and Estiarte 1998), a recent meta-analysis showed nearly equal number of studies reporting increases and decreases in nitrogen-rich compounds such as amino acids and nucleosides (Sardans et al. 2020). Together, these findings indicate that metabolic responses to elevated CO_2_ are highly compound-specific and depend strongly on species identity, developmental stages, and environmental context.

Despite the growing number of studies, the combined effects of elevated CO_2_, water availability and nitrogen supply remain insufficiently resolved, particularly for trees under semi-natural or field conditions. The need for such multifactorial experiments has been highlighted by Calfapietra et al. (2010), who emphasized that elevated CO_2_ should be studied together with interacting climate-change factors to improve predictions of forest responses. This is particularly relevant for interactions among CO_2_, water and nitrogen, because drought and nitrogen limitation influence different but tightly connected components of the plant carbon economy. Water deficit constrains stomatal conductance, leaf water status and carbon uptake, whereas nitrogen limitation restricts investment in photosynthetic proteins, sink activity and the metabolic use of newly assimilated carbon. Previous studies on spruce and beech (Košvancová et al. 2009, Kolář et al. 2017, Ofori-Amanfo et al. 2021, 2023) demonstrated that the CO_2_ fertilization effect on photosynthesis and growth diminishes, or may disappear, under limited water or nitrogen supply. Given the ecological importance of saplings for forest regeneration, understanding how young trees respond to rising CO_2_ under resource limitations is critical, particularly in temperate Central European mountain forests, where nitrogen availability is naturally low and summer droughts are intensifying (Körner 2022).

In this study, we investigated how elevated CO_2_, water availability and nitrogen supply interact to shape physiological and metabolic responses in two ecologically and functionally contrasting tree species, coniferous Norway spruce (*Picea abies* (L.) Karst.) and broadleaved European beech (*Fagus sylvatica* L.). These species differ in drought-regulation strategy: the more isohydric Norway spruce tends to maintain relatively stable leaf water potential through earlier stomatal closure, while the more anisohydric European beech keeps stomata open longer, thereby sustaining CO_2_ uptake but increasing water loss and exposure to stress during prolonged drought (Ulrich and Grossiord 2023). We therefore expected elevated CO_2_ to exert the dominant influence on both physiology and metabolism, while its effects would be strongly modified by water and nitrogen availability. More specifically, we expected beech to show greater physiological and metabolic plasticity under elevated CO_2_, but also greater susceptibility to photosynthetic downregulation, accompanied by resource-dependent shifts in soluble sugars, amino acids and organic acids. By contrast, we expected spruce to maintain a more conservative response, with comparatively stable photosynthetic capacity and stronger investment in phenolic-based defence metabolism across resource conditions. To test these expectations, we addressed the following hypotheses:

1. Elevated atmospheric CO_2_ exerts a dominant influence on tree physiology and metabolism across species, but the magnitude of this response is strongly modulated by water and nitrogen availability.
2. Because more anisohydric beech and more isohydric spruce differ in stomatal regulation and metabolic plasticity, they exhibit contrasting acclimation strategies to elevated CO_2_ under resource limitation, with beech showing stronger physiological and metabolic adjustment and spruce maintaining more stable photosynthetic capacity and defence metabolism.
3. Species-specific regulation of photosynthetic capacity under elevated CO_2_ is linked to contrasting shifts in carbon and nitrogen metabolism, with stronger photosynthetic downregulation and metabolic adjustment expected in beech than in spruce.

## Materials and methods

### Plants and experimental design

The experiment was conducted at the ecological research station Bílý Kříž, part of the AnaEE-ERIC European Research Infrastructure Consortium, in the Moravian-Silesian Beskydy Mountains, Czech Republic (49°30′09″ N, 18°32′20″ E; 908 m a.s.l.). The site has a cool and humid climate, with a mean annual air temperature of 6.7 °C, a mean relative air humidity of 80%, and a mean annual precipitation of 1374 mm (2010–20 average). Due to frequent cloud cover, ~74% of incident photosynthetically active radiation (PAR) is below 500 μmol photons m^−2^ s^−1^. Seasonal courses of air temperature, relative humidity and PAR in 2021 (the study year) are shown in Figure S1 available as Supplementary Data at *Tree Physiology* Online. The site is characterized by acidic, nutrient-poor podzolic soils typical of mountain forest ecosystems. The soil at Bílý Kříž is classified as Haplic Podzol (WRB), developed on Mesozoic Godula sandstone (flysch type), with low pH and low base status. Published deposition data indicate background nitrogen inputs of ~9.2 kg N ha^−1^ year^−1^ in bulk precipitation and 13.1 kg N ha^−1^ year^−1^ in throughfall. A detailed site description is provided in Urban et al. (2001) and Ofori-Amanfo et al. (2021, 2023).

In 2019, European beech (*Fagus sylvatica* L.) and Norway spruce (*Picea abies* (L.) Karst.) saplings (4-year-old; ~0.5 m tall; sourced from a forestry nursery) were planted into the native soil. Beginning in May 2020, saplings were exposed to either ambient (~400 μmol CO_2_ mol^−1^; hereafter AC) or elevated (~700 μmol CO_2_ mol^−1^; hereafter EC) atmospheric CO_2_ concentrations using two lamellar domes (Urban et al. 2001), one maintained at AC and one at EC. CO_2_ enrichment was maintained continuously from May to the end of September 2020 and was suspended during winter dormancy. Fumigation resumed on 1 May 2021, before budburst, and continued until the measurement and sampling campaign in the second half of August 2021. Thus, saplings had received ~250 days of cumulative exposure to their respective CO_2_ treatments over two growing seasons before the measurements reported here.

The CO_2_ treatment was applied at the whole-dome level. Each dome (10 × 10 m) was divided into three spatial blocks. Within each block, the four combinations of water availability and nitrogen supply were randomly assigned to individual plots (Figure 1). Each water × nitrogen combination was represented once per block and therefore replicated three times within each CO_2_ environment. To prevent lateral soil water movement between plots, PVC boards (5 mm thickness) were inserted vertically to a depth of ~50 cm, reaching the weathering bedrock.

**Figure 1:**
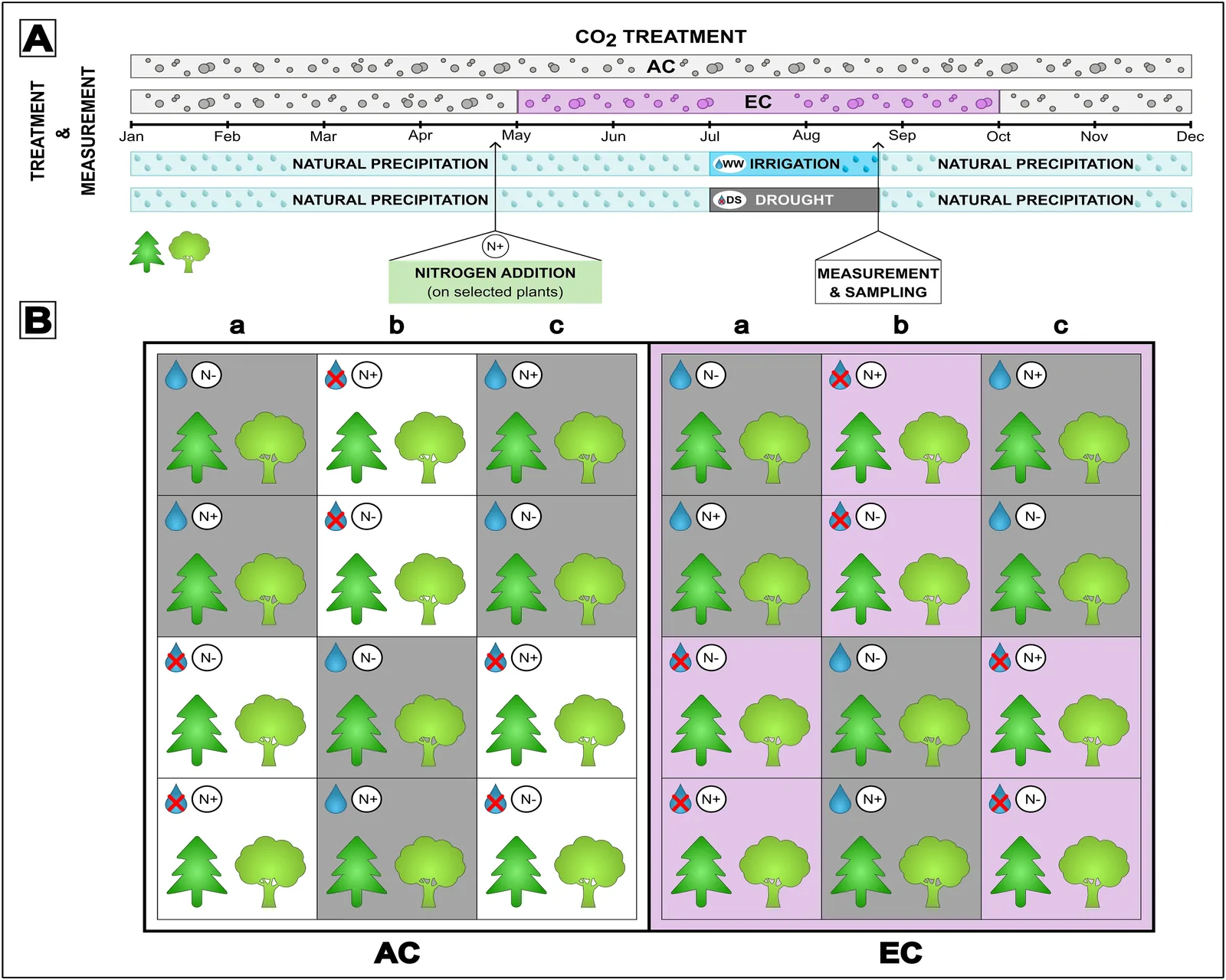
Experimental design and treatment chronology. (A) Seasonal timing of the CO_2_ treatment, nitrogen addition, water treatments, and the measurement and sampling period. Saplings were grown in lamellar domes under AC or EC concentrations. CO_2_ treatment was applied continuously from May to the end of September 2020 and again from May to the second half of August 2021, when measurements and sampling were conducted. Fumigation was suspended during winter dormancy. Nitrogen addition was applied once in late April (N^+^; calcium nitrate, 60 kg N ha^−1^), before beech budburst and the onset of current-year shoot growth in spruce; control plots received no nitrogen addition (N^−^). Outside the drought-treatment period, open lamellae allowed natural precipitation to enter the domes. During the drought treatment (early July to mid-August), the lamellae were closed to exclude precipitation; well-watered (WW) plots received 20 mm of irrigation per week, whereas drought-stressed (DS) plots received no irrigation. (B) Plot layout within each CO_2_ treatment. Lowercase letters (a–c) indicate the three spatial blocks within each dome; within each block, plots received the four WW/DS × N^+^/N^−^ treatment combinations, so each water × nitrogen combination was represented once per block (three plot-level replicates within each CO_2_ environment).

Within each block, four treatment combinations were established in a 2 × 2 factorial design with two levels of water availability [well-watered (WW) vs drought-stressed (DS)] and nitrogen supply [nitrogen addition (N^+^) vs no nitrogen addition (N^−^)]. During normal operation, the automatically controlled lamellae were kept open whenever conditions allowed, maximizing exchange between the dome and outside atmosphere and permitting natural precipitation to enter the experimental area. During the 5-week drought-treatment period in both 2020 and 2021, from early July until the measurement campaign in the second half of August, the lamellae of both domes were kept closed to exclude natural precipitation. During this period, WW plots received 20 mm of irrigation per week, whereas DS plots received no irrigation. Nitrogen was supplied as calcium nitrate at 60 kg N ha^−1^ to N^+^ plots, whereas N^−^ plots received no additional nitrogen. This dose was chosen to provide a clear experimental contrast above ambient annual nitrogen input, being ~4.6 times higher than the annual throughfall nitrogen flux at the site, and to alleviate nitrogen limitation in the fertilized plots. Nitrogen was applied once in late April of each year, before beech budburst and the onset of current-year shoot growth in spruce. Each plot contained 12 saplings per species.

The water treatment produced clear differences in soil moisture during the drought period: volumetric soil water content ranged from 19 to 23% under DS and from 21 to 30% under WW conditions (Figure S2 available as Supplementary Data at *Tree Physiology* Online), as measured with CS616L sensors installed at 5 cm depth (Campbell Scientific, Logan, UT, USA). Based on the observed reduction in soil moisture and the corresponding leaf water potential responses measured at the end of the drought-treatment period (Figure S3 available as Supplementary Data at *Tree Physiology* Online; see ``Leaf water status and foliar stoichiometry''), the treatment represented moderate drought relative to site conditions, with a stronger physiological effect in beech than in spruce.

All physiological measurements and metabolite sampling reported here were conducted in the second half of August 2021, after two consecutive growing seasons (2020 and 2021) under the combined CO_2_, water and nitrogen treatments. The drought treatment and nitrogen addition were applied in both years.

### Physiological and gas-exchange measurements

For each species and treatment plot, three saplings were randomly selected from the 12 saplings present and used for the leaf- and shoot-level physiological measurements and metabolite sampling reported here. These saplings were treated as subsamples within each plot, and their mean was used as the plot-level value for subsequent statistical analyses. Thus, the experimental unit for physiological and biochemical analyses was the plot rather than the individual sapling. In spruce, measurements were performed on current-year shoots. Except for predawn water-potential measurements, physiological measurements were carried out between 10:00 and 15:00 h local time on healthy intact leaves or shoots in the upper third of each sapling to minimize shading effects.

Water potential (Ψ) was measured at pre-dawn (05:00 h) and midday (12:00 h) using a Scholander-type pressure chamber (Model 1000, PMS Instrument Company, Albany, OR, USA). Gas exchange parameters, including net CO_2_ assimilation rate (*A*), stomatal conductance (*G*s) and transpiration rate (*E*), were measured using a LI-6800 portable photosynthesis system (LI-COR Biosciences, Lincoln, NE, USA). During measurements, leaf temperature was maintained at ~25 °C and vapour pressure deficit (VPD) 1.1–1.9 kPa. Light-saturated *A*, *G*s and *E* values were recorded after 3–5 min of stabilization at a PAR of 1400 μmol photons m^−2^ s^−1^ and at the respective growth CO_2_ concentrations (400 μmol CO_2_ mol^−1^ for AC and 700 μmol CO_2_ mol^−1^ for EC plants). Instantaneous light-saturated water-use efficiency (WUE) was calculated as *A*/*E* (Ofori-Amanfo et al. 2023).

After these measurements, CO_2_ response curves (*A*–*C*i) were obtained at external CO_2_ concentrations of 1500, 700, 400, 250, 150 and 75 μmol CO_2_ mol^−1^. The maximum rate of Rubisco carboxylation (*V*_Cmax_) and maximum electron transport rate (*J*_max_) were derived from these curves using the Photosyn Assistant software v1.1.2 (Dundee Scientific, Dundee, UK), based on the Farquhar et al. (1980) model.

In addition, a Multiplex fluorescence sensor (Force-A, Orsay, France) was used to measure UV-excited blue fluorescence (BF_UV) in fully expanded beech leaves and current-year spruce needles. The sensor was positioned 2–4 cm from the leaf or needle surface, and measurements were taken while avoiding direct sunlight on the sensor head.

### Metabolomic profiling

Leaf (beech) and needle (spruce) samples (~500 mg fresh weight) were collected around midday (11:00–13:00 h local time) to minimize diurnal variability in metabolite composition. Samples were immediately frozen in liquid N_2_, transported to the laboratory, lyophilized, and stored at −80 °C until analysis. For extraction, 100 mg of lyophilized tissue was homogenized and subsequently extracted using a methanol:chloroform:water solution (1:2:2, v/v/v) to yield a broad range of primary and secondary metabolites. After phase separation, aliquots of the upper polar phase were collected and analysed.

Metabolites were profiled using an UltiMate 3000 high-performance liquid chromatography (HPLC) system coupled to an LTQ Orbitrap XL high-resolution mass spectrometer (HRMS; Thermo Fisher Scientific, Waltham, MA, USA) equipped with a heated electrospray ionization (HESI II) source. The Orbitrap HRMS was operated in full-scan mode at a resolution of 60,000 over *m*/*z* 50–1000 in positive ionization mode and *m*/*z* 65–1000 in negative ionization mode. Instrument performance was monitored using phenolic standards analysed after every 25 samples. Lock masses (phthalates) were used to verify mass accuracy, and blank samples were included periodically for quality control.

Compounds were identified by matching spectra to a mass-spectral library constructed from analytical standards analysed in MS and MS^n^ modes. Chromatographic separation was performed on a Hypersil GOLD column (Thermo Scientific, USA; 150 mm × 2.1 mm, 3 μm film thickness). The mobile-phase flow rate was set to 0.3 mL min^−1^, and the column temperature was maintained at 30 °C. The mobile phase consisted of acetonitrile and water with 0.1% acetic acid. Both solvents were filtered and degassed in an ultrasonic bath for 10 min before use. Gradient elution started at 10% acetonitrile and 90% water (maintained for 5 min), followed by a linear increase to 90% acetonitrile over the next 15 min. This composition was maintained for 5 min, after which a 5-min equilibration returned the system to initial conditions. UV absorbance was monitored at 254, 272, 274 and 331 nm throughout the chromatographic run. Further methodological details are provided in Večeřová et al. (2016).

### Statistical data processing

Statistical analyses were performed separately for each species using plot-level means. For each species and treatment plot, values from the three measured saplings were averaged, and the resulting plot mean was used as the unit of analysis. Thus, three plot-level replicates were available for each water × nitrogen treatment combination within each CO_2_ environment.

Multifactorial analysis of variance (ANOVA) was used to evaluate the main effects of CO_2_ concentration (AC vs EC), water availability (WW vs DS) and nitrogen supply (N^+^ vs N^−^), all two-way interactions (CO_2_ × WA, CO_2_ × NS, WA × NS) and the three-way interaction (CO_2_ × WA × NS). Block was included as a blocking factor nested within the CO_2_ environment to account for spatial variation among the three blocks within each dome. Because the CO_2_ treatment was applied at the whole-dome level, with one dome maintained at AC and one at EC, it was not independently replicated at the chamber level. Accordingly, effects associated with CO_2_, and interactions involving CO_2_, should be interpreted with appropriate caution as treatment patterns observed within this experimental setup rather than as fully replicated tests of an independent CO_2_ treatment. When ANOVA indicated significant effects (*P* ≤ 0.05), differences among treatment means were assessed using Tukey’s HSD post-hoc test.

ANOVA and post hoc analyses were performed in STATISTICA 12 (StatSoft, Tulsa, CA, USA). Prior to principal component analysis (PCA), variables were mean-centred and scaled to unit variance to ensure equal weighting of variables measured on different scales. PCA ordinations were computed in Canoco 5 (Microcomputer Power, Ithaca, NY, USA). Boxplots, PCA biplots, and polar clustering heatmaps were prepared in OriginPro 2021b (OriginLab, Northampton, MA, USA).

## Results

### Leaf water status and foliar stoichiometry

The experimental treatments affected leaf water status and foliar stoichiometry (Figures S3–S4 available as Supplementary Data at *Tree Physiology* Online). In beech, DS plants had more negative midday leaf water potential (Ψ_noon_) than well-watered (WW) plants under both AC and EC (Figure S3 available as Supplementary Data at *Tree Physiology* Online). In spruce, Ψ_noon_ was comparatively stable across treatments, indicating only mild water stress. In both species under EC × DS, N^+^ was associated with higher (less negative) Ψ_noon_ values than under N^−^, indicating partial buffering of drought effects.

Foliar stoichiometry also differed markedly between the two species, with beech showing higher nitrogen content and lower C:N ratios than spruce across treatments (Figure S4 available as Supplementary Data at *Tree Physiology* Online). In beech, nitrogen supply increased foliar nitrogen content, whereas no corresponding nitrogen effect was detected in spruce. Elevated CO_2_ reduced foliar nitrogen content and increased C:N in both species, with the change being more pronounced in spruce. Water availability had no significant effect on either stoichiometric variable, and nitrogen supply did not significantly affect C:N in either species. Thus, EC was the most consistent driver of foliar stoichiometric responses, particularly in spruce.

### Multivariate responses of physiological and biochemical traits

Principal component analysis, conducted separately for beech and spruce, revealed that the CO_2_ treatment was most strongly associated with variation along PC1, which explained 32.7% of the total variance in beech and 27.9% in spruce (Figure 2). In both species, EC was positively associated with light-saturated CO_2_ assimilation rate (*A*), Ψ_noon_ and WUE, and negatively associated with stomatal conductance (*G*s) and transpiration (*E*). In beech, EC was also negatively associated with *V*_Cmax_ and *J*_max_, consistent with photosynthetic downregulation. In spruce, *V*_Cmax_ and *J*_max_ showed no consistent association with EC, although *J*_max_ tended to be slightly higher under EC. Elevated CO_2_ was also associated with higher C:N ratios and lower foliar nitrogen in both species.

**Figure 2:**
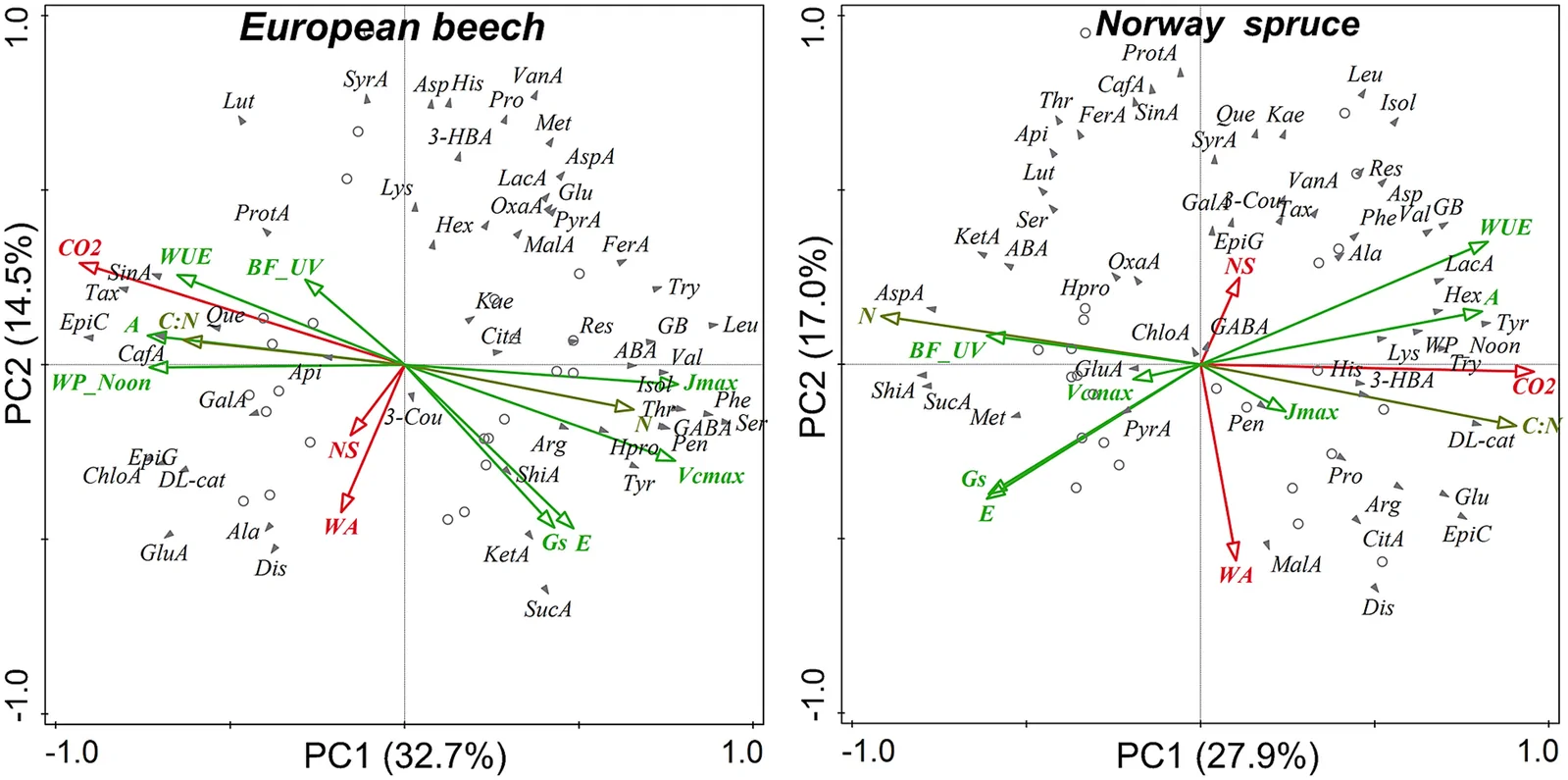
PCA biplots showing effects of CO_2_ concentration (CO2), water availability (WA) and nitrogen supply (NS) on physiological and biochemical traits in European beech (left) and Norway spruce (right). Red vectors represent the experimental factors; their direction and length indicate the direction and relative strength of their association with the PCA ordination. Physiological variables (light green): A (light-saturated CO_2_ assimilation rate), Gs (stomatal conductance), E (transpiration rate), WUE (water-use efficiency; A/E), WP_Noon (midday leaf water potential), Vcmax (maximum Rubisco carboxylation capacity), Jmax (maximum electron transport rate), BF_UV (UV-excited blue fluorescence index). Stoichiometric parameters (khaki): N (foliar nitrogen content per dry mass unit), C:N (foliar carbon-to-nitrogen ratio). Metabolites detected in the targeted metabolomic analysis (black triangles): Phenolics—3-HBA (3-hydroxybenzoic acid), ProA (protocatechuic acid), 3-Cou (3-coumaric acid), VanA (vanillic acid), GalA (gallic acid), CafA (caffeic acid), FerA (ferulic acid), SyrA (syringic acid), SinA (sinapic acid), ChloA (chlorogenic acid), Res (resveratrol), Api (apigenin), Lut (luteolin), Kae (kaempferol), Que (quercetin), EpiC (epicatechin), EpiG (epigallocatechin), DL-cat (DL-catechin), Tax (taxifolin). Saccharides—Pen (pentoses), Hex (hexoses), Dis – disaccharides. Amino acids—Ala (alanine), Val (valine), Leu (leucine), Ileu (isoleucine), Ser (serine), Thr (threonine), Met (methionine), Lys (lysine), AspA (aspartic acid), Asp (asparagine), GluA (glutamic acid), Glu (glutamine), Arg (arginine), His (histidine), Phe (phenylalanine), Try (tryptophan), Pro (proline), Tyr (tyrosine), Hpro (hydroxy proline). TCA/shikimate-related acids— KetA (α-ketoglutaric), PyrA (pyruvic), ShiA (shikimic), CitA (citric), MalA (malic), SucA (succinic), OxaA (oxaloacetic) and LacA (lactic). Stress-related compounds—ABA (abscisic acid), GB (glycine betaine), GABA (γ-aminobutyric acid).

UV-excited blue fluorescence (BF_UV), a proxy for phenolic-related compounds, was negatively associated with EC in spruce but slightly positively associated in beech, indicating contrasting phenolic responses between the two species (Figure S3 available as Supplementary Data at *Tree Physiology* Online). Consistent with this pattern, beech under EC showed higher contents of several phenolics (e.g. sinapic acid, taxifolin, epicatechin, quercetin and caffeic acid), whereas in spruce several phenolics were associated primarily with drought, with additional modulation by nitrogen supply, rather than showing a consistent association with EC alone.

Amino-acid profiles were also species-specific. In beech, EC was associated with lower contents of numerous amino acids (e.g. phenylalanine, serine, leucine, isoleucine, valine, threonine) and with reduced contents of glycine betaine (GB), γ-aminobutyric acid (GABA) and abscisic acid (ABA). In spruce, EC was negatively associated with aspartic acid but positively associated with tyrosine and tryptophan; GB showed a slight positive association with EC, whereas GABA was largely unaffected.

PC2 primarily captured variation associated with water availability, explaining 14.5% and 17.0% of the variance in beech and spruce, respectively. Higher water availability was associated with increases in a limited set of metabolites, including disaccharides and selected amino acids (e.g. alanine, glutamic acid) in beech, and TCA-cycle metabolites (e.g. malic and citric acids) in spruce. By contrast, drought was associated with broader species-specific shifts. In beech, these included higher contents of phenolic acids (syringic, vanillic, 3-hydroxybenzoic) and amino acids (e.g. asparagine, proline, histidine, methionine), whereas in spruce they included higher contents of several phenolics (e.g. protocatechuic, caffeic, sinapic, ferulic and syringic acids (SyrA), quercetin, kaempferol and apigenin).

Nitrogen supply was also associated with variation along PC2, although less strongly than EC or water availability, and the pattern differed between species. In beech, N^−^ was associated with higher contents of several metabolites that were also associated with drought, including selected phenolic acids (vanillic and syringic) and amino acids (asparagine, proline, methionine and histidine). In spruce, by contrast, a similar drought-associated metabolite pattern was linked to N^+^, particularly for phenolics (e.g. quercetin, kaempferol and SyrA) and branched-chain amino acids (leucine, isoleucine and valine).

A more detailed overview is provided by the polar heatmaps, in which hierarchical clustering groups traits according to similarity in their treatment-response profiles based on Pearson’s correlation coefficients, while treatment combinations are displayed in a fixed order (Figure 3). In beech, a large group of parameters decreased under EC, including *E*, *G*s, *V*_Cmax_, *J*_max_, foliar nitrogen, several amino acids (e.g. Thr, Tyr, Arg, Leu, Ileu, Ser, Phe, Try) and TCA-cycle intermediates (e.g. succinic acid (SucA), KetA). Conversely, EC increased *A*, WUE, Ψ_noon_ and several phenolics (e.g. EpiC, SinA, ChloA, CafA, Que, Lut, ProA). Under AC × DS × N^−^, beech showed higher contents of protective compounds, including GB, GABA, ABA and Kae, whereas under EC × DS × N^−^ beech accumulated stress-related amino acids (Pro, His, Asp, Met, AspA) together with SyrA.

**Figure 3:**
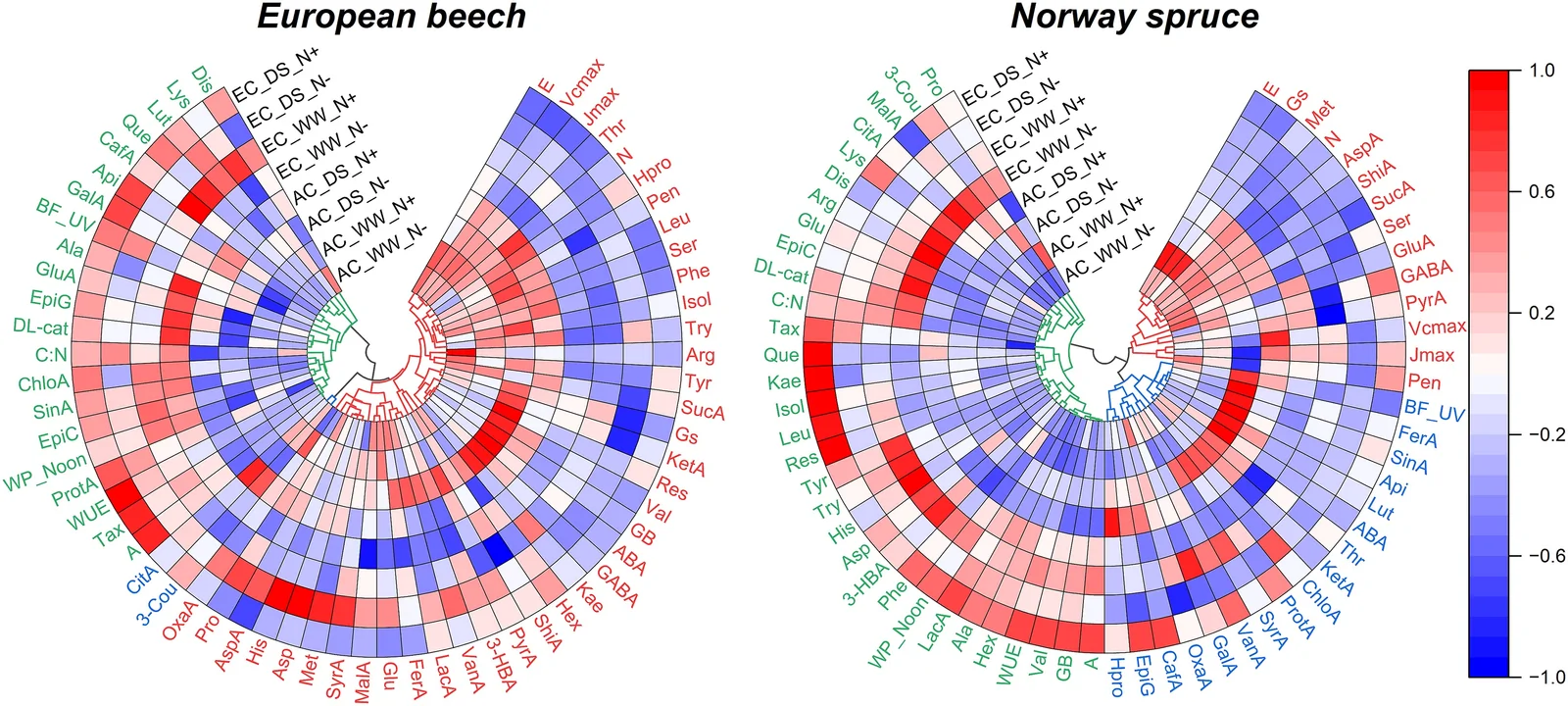
Two-dimensional polar heatmaps with hierarchical clustering summarizing responses of physiological and biochemical traits to CO_2_ treatment (AC, EC), water availability (WW, DS) and nitrogen supply (N^+^, N^−^) in European beech (left) and Norway spruce (right). The central dendrogram shows hierarchical clustering of measured traits based on Pearson’s correlation coefficient. Treatment combinations are displayed in a fixed and identical order for both species and were not included in the clustering. Colour scale: red indicates values above and blue values below the cross-treatment mean; darker shades indicate larger deviations.

In spruce, lower values under EC were observed for *E*, *G*s, foliar nitrogen, shikimic acid (ShiA), SucA and serine. By contrast, higher values under EC were observed for *A*, WUE, hexoses, alanine, valine, phenylalanine, GB, taxifolin and lactic acid (Figure 3). Under AC × DS × N^−^, higher contents were observed for ferulic, sinapic, and chlorogenic acids, ABA, apigenin, luteolin, threonine and α-ketoglutaric acid. Under EC × DS × N^−^, the highest contents were observed for malic and citric acids, lysine, arginine, glutamine, epicatechin, DL-catechin and disaccharides.

### Gas-exchange parameters

#### Light-saturated CO_2_ assimilation rate (*A*), stomatal conductance (*G*s) and water-use efficiency

Across treatments, EC increased *A* and water-use efficiency (WUE) and reduced *G*s, with water and nitrogen availability modulating these responses in a species-specific manner (Figure 4). In beech, the main effect of CO_2_ on *A* was significant, with additional CO_2_ × WA and CO_2_ × NS interactions. Post hoc tests showed that EC significantly increased *A* under DS at N^+^. For *G*s, the main effect of CO_2_ was significant, as were the CO_2_ × WA and CO_2_ × NS interactions. Post-hoc tests showed a significant EC-induced reduction in *G*s under DS × N^−^, whereas WA and NS alone had no significant effects on *G*s. WUE was significantly affected by CO_2_ and WA; EC increased WUE, particularly under DS and/or N^+^.

**Figure 4:**
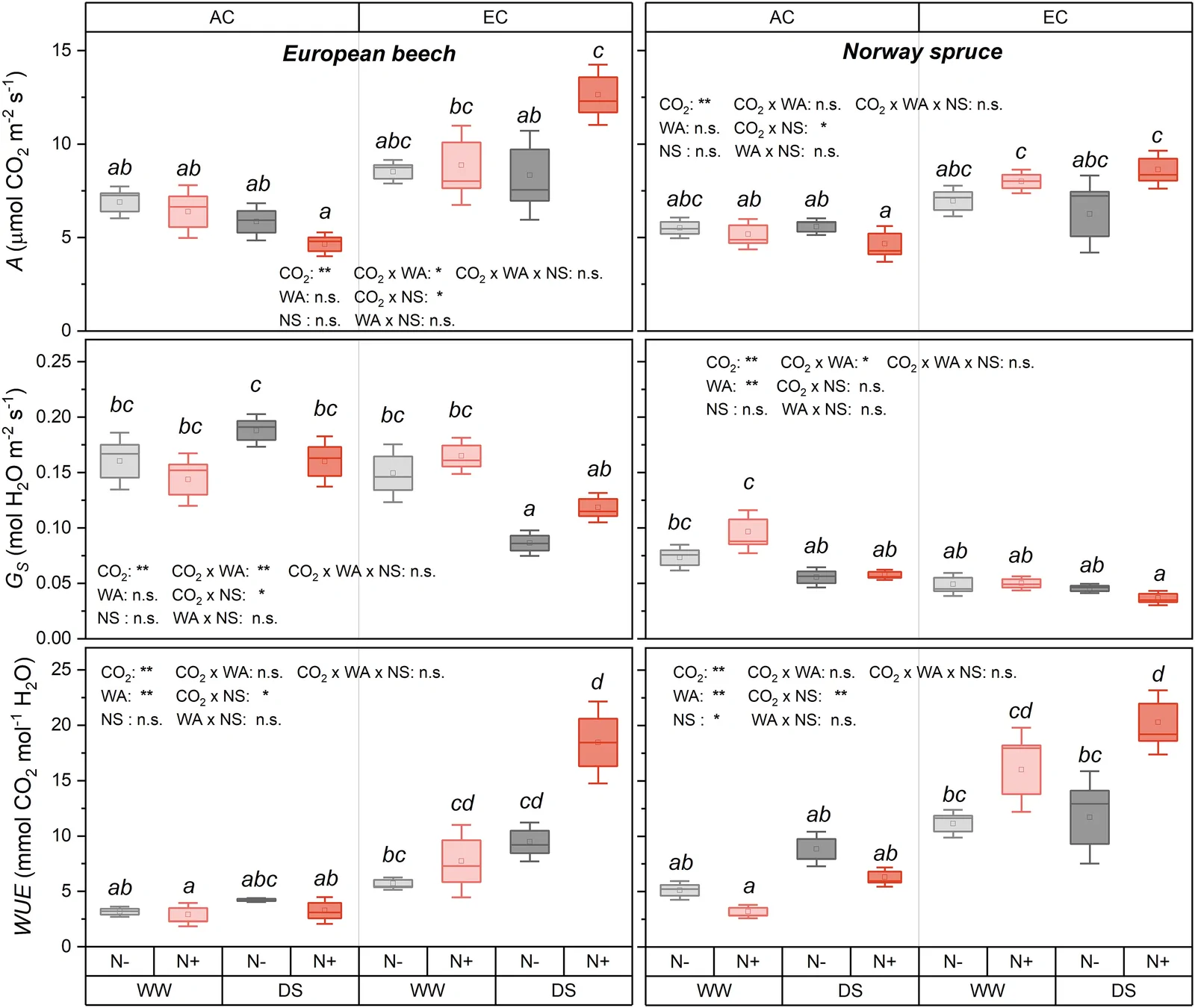
Boxplots showing effects of CO_2_ concentration (CO_2_), nitrogen supply (NS) and water availability (WA) on light-saturated CO_2_ assimilation rate (*A*), stomatal conductance (*G*_S_) and water-use efficiency (WUE) in European beech (left) and Norway spruce (right). Gas-exchange measurements were performed at the respective growth CO_2_ concentrations at the end of the 5-week water-limitation period in the second half of August. AC—ambient CO_2_ concentration (~400 μmol CO_2_ mol^−1^); EC—elevated atmospheric CO_2_ concentration (~700 μmol CO_2_ mol^−1^); WW—well-watered (20 mm irrigation per week during the drought-treatment period); DS—drought-stressed (no irrigation during the drought-treatment period); N^+^—nitrogen addition; N^-^—no nitrogen addition. Boxes show the 25th–75th percentiles, the horizontal lines indicate medians, points indicate means, and whiskers extend to 1.5× IQR. Different letters indicate significant differences among treatment combinations based on Tukey HSD tests (*P* ≤ 0.05). Asterisks indicate significant ANOVA main effects or interactions (^*^P < 0.05, ^**^P < 0.01); ‘n.s.’ indicates non-significant effects.

In spruce, CO_2_ and the CO_2_ × NS interaction significantly affected *A*, with EC increasing *A* under N^+^ at both water levels. *G*s was significantly affected by CO_2_, WA and the CO_2_ × WA interaction, with EC significantly reducing *G*s under WW × N^+^. WUE was significantly influenced by CO_2_, WA and NS, with a significant CO_2_ × NS interaction. Elevated atmospheric CO_2_ increased WUE under N^+^, with the highest values observed under DS.

#### Maximum carboxylation (*V*_Cmax_) and electron transport rates (*J*_max_)

In beech, CO_2_ had a significant main effect on both *V*_Cmax_ and *J*_max_. Exposure to EC resulted in lower *V*_Cmax_ and *J*_max_ across water and nitrogen treatments, with no significant effects of WA, NS or their interactions with CO_2_ (Figure 5).

**Figure 5:**
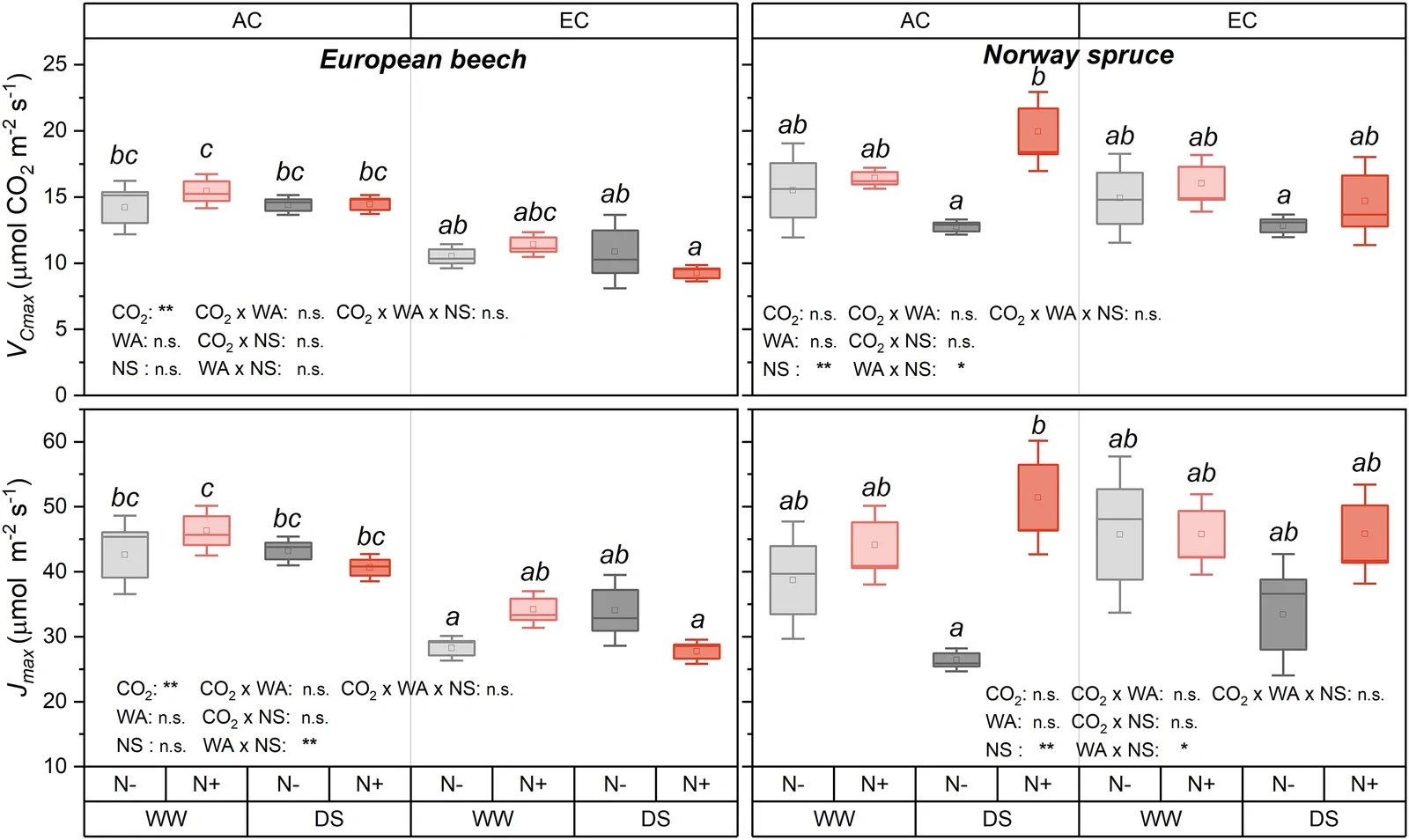
Boxplots showing effects of CO_2_ concentration (CO_2_), nitrogen supply (NS) and water availability (WA) on the maximum rate of Rubisco carboxylation (*V*_Cmax_) and maximum electron transport rate (*J*_max_) in European beech (left) and Norway spruce (right). *V*_Cmax_ and *J*_max_ were derived from *A*–*C*i response curves measured at the end of the 5-week water-limitation period in the second half of August. See Figure 4 for plotting conventions, treatment abbreviations and ANOVA notation. Different letters indicate significant differences among treatment combinations based on Tukey HSD tests (*P* ≤ 0.05). Asterisks indicate significant ANOVA main effects or interactions (^*^P < 0.05, ^**^P < 0.01); ‘n.s.’ indicates non-significant effects.

In spruce, *V*_Cmax_ and *J*_max_ were significantly affected by nitrogen supply (NS) and the WA × NS interaction. Drought reduced both *V*_Cmax_ and *J*_max_ under N^−^, whereas N^+^ buffered this negative drought effect. This pattern was most apparent under AC conditions (Figure 5).

### Species-specific metabolic adjustments

To illustrate compound-specific metabolic responses, we focused on selected metabolites with high PCA loadings and contrasting responses to the experimental factors. In both species, the selected TCA- and shikimate-pathway metabolites were significantly affected by CO_2_. However, additional effects of water availability, nitrogen supply, and their interactions were evident mainly in beech, whereas in spruce CO_2_ was the only significant factor for the metabolites examined (Figure 6). In beech, SucA was affected by CO_2_, NS and the WA × NS interaction and generally declined under EC, with higher SucA under EC × DS × N^+^ than EC × DS × N^−^. Shikimic acid was affected by CO_2_ and NS, with significant CO_2_ × WA, WA × NS and CO_2_ × WA × NS interactions. In spruce, SucA and ShiA contents were comparatively stable across resource treatments, with CO_2_ being the only significant factor for both metabolites.

**Figure 6:**
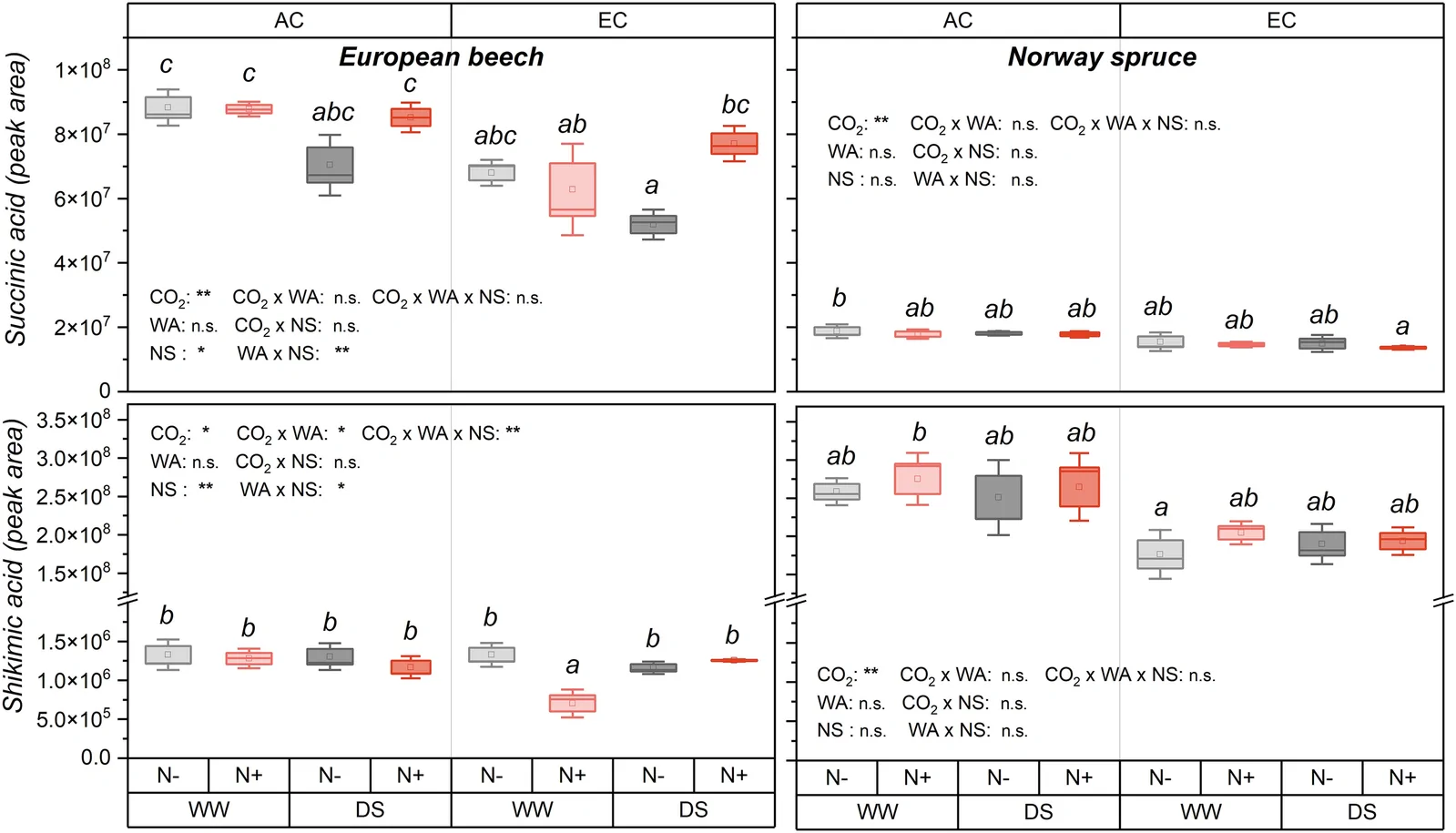
Boxplots showing effects of CO_2_ concentration (CO_2_), nitrogen supply (NS) and water availability (WA) on succinic acid (SucA) and shikimic acid (ShiA) contents in European beech (left) and Norway spruce (right). Measurements were performed at the end of the five-week water-limitation period in the second half of August. See Figure 4 for plotting conventions, treatment abbreviations, and ANOVA notation. Different letters indicate significant differences among treatment combinations based on Tukey HSD tests (*P* ≤ 0.05). Asterisks indicate significant ANOVA main effects or interactions (^*^P < 0.05, ^**^P < 0.01); ‘n.s.’ indicates non-significant effects.

Amino-acid responses also differed between species (Figure S5 available as Supplementary Data at *Tree Physiology* Online). In beech, phenylalanine content decreased markedly under EC and was significantly affected by CO_2_, WA, NS and the WA × NS interaction, whereas leucine content was affected by CO_2_, WA and NS, and generally decreased under EC. In spruce, phenylalanine was affected only by CO_2_, whereas leucine was significantly affected by CO_2_, WA, NS and the WA × NS interaction, with the highest contents under EC × DS × N^+^. Soluble carbohydrates showed a different pattern (Figure S6 available as Supplementary Data at *Tree Physiology* Online). Hexose contents in beech leaves were generally high but unaffected by the experimental factors, whereas in spruce they were comparatively lower and increased significantly under EC. Disaccharides in beech were affected only by the WA × NS interaction, while in spruce, they were affected by CO_2_, WA, NS and the WA × NS interaction, with the highest contents under EC × WW × N^−^.

Responses of phenolic compounds were strongly species- and compound-specific (Figure S7 available as Supplementary Data at *Tree Physiology* Online). Epicatechin contents were higher under EC in both species; in beech, epicatechin was affected by CO_2_ and NS, whereas in spruce significant effects of CO_2_, WA and the CO_2_ × WA and CO_2_ × NS interactions were detected. Caffeic acid showed stronger treatment dependence. In beech, CO_2_, WA, NS, all pairwise interactions, and the CO_2_ × WA × NS interaction had significant effects, with the highest contents under EC × WW × *N*^+^. In spruce, caffeic acid was affected by CO_2_, WA and NS, with significant CO_2_ × NS and WA × NS interactions, and reached its highest contents under EC × DS × *N*^+^.

Stress-related metabolites further differentiated the two species (Figure S8 available as Supplementary Data at *Tree Physiology* Online). Absolute abscisic acid (ABA) contents were substantially lower in beech than in spruce under both CO_2_ treatments. In beech, ABA was significantly affected by CO_2_ and WA, with significant CO_2_ × WA, CO_2_ × NS and WA × NS interactions. A marked decline occurred under EC × DS × N^−^ relative to AC × DS × N^−^. In spruce, CO_2_, WA, NS and CO_2_ × NS had significant effects on ABA. Drought strongly increased ABA under AC, particularly at N^−^, whereas ABA was comparatively stable under EC, with only minimal additional effects of WA and NS. Glycine betaine (GB) content in beech was significantly affected by CO_2_, WA and all pairwise interactions, but varied only little among resource treatments under EC. In spruce, GB contents remained uniformly low across treatments, with only a small but significant increase under EC.

## Discussion

This study addressed three interrelated hypotheses: (1) elevated atmospheric CO_2_ exerts a dominant influence on tree physiology and metabolism, while the magnitude of this response is modulated by water and nitrogen availability; (2) the more anisohydric beech and more isohydric spruce exhibit contrasting acclimation strategies to elevated CO_2_, with greater physiological sensitivity and metabolic plasticity in beech and greater stability of photosynthetic capacity and defence metabolism in spruce; and (3) species-specific regulation of photosynthetic capacity under elevated CO_2_ is associated with contrasting shifts in carbon and nitrogen metabolism, with stronger photosynthetic downregulation and metabolic adjustment in beech than in spruce.

### Dominant effects of elevated CO_2_ and their modulation by resources

A central outcome of this study is that elevated CO_2_ did not simply stimulate carbon gain in both species, but also revealed contrasting capacities to maintain photosynthetic functioning under differing water and nitrogen availability. The stimulatory effects of elevated atmospheric CO_2_ (EC) on C_3_ plant physiology and growth are well documented (Urban 2003, Ainsworth and Rogers 2007, Poorter et al. 2022). Our results broadly agree with this framework, as EC was the strongest multivariate driver of physiological (e.g. *A*, *G*s, *E*, WUE) and metabolic (e.g. amino acid and phenolic profiles) variation (Figure 2). However, the magnitude, and for some responses also the direction, of EC effects depended on resource availability, indicating that CO_2_-driven enhancement of carbon gain was not expressed equally across different combinations of water and nitrogen availability.

In beech, the response of light-saturated CO_2_ assimilation to EC depended strongly on resource availability. The greatest stimulation of *A* occurred under drought when nitrogen was supplied (EC × DS × N^+^; Figure 4), indicating that sufficient nitrogen availability allowed beech to benefit from EC even under reduced water availability. By contrast, under DS × N^−^, EC did not significantly enhance *A* relative to AC. Despite these contrasting responses of *A*, EC reduced *V*_Cmax_ and *J*_max_ across resource conditions (Figure 5), indicating photosynthetic downregulation. This apparent decoupling indicates that reduced photosynthetic capacity under EC did not necessarily translate into lower assimilation measured at the growth CO_2_ concentration, particularly when nitrogen was available. Such responses are consistent with evidence that water and nitrogen availability strongly regulate the extent of photosynthetic stimulation and feedback acclimation under elevated CO_2_ (Luo et al. 1999, Reich et al. 2006, Norby et al. 2010, Jin et al. 2018, Ainsworth and Long 2021). In contrast, spruce maintained comparably stable *V*_Cmax_ and *J*_max_ across AC and EC and across water and nitrogen treatments, indicating greater stability of biochemical carboxylation and electron-transport capacity. Elevated CO_2_ (EC)-induced photosynthetic downregulation in conifers has been particularly reported in older needles with low sink activity (Urban et al. 2012), suggesting that needle age and sink strength may influence the extent of biochemical acclimation to long-term EC.

In both species, EC increased WUE, but the associated gas-exchange responses differed. Because WUE was calculated as *A*/*E*, this response reflects the balance between carbon gain and transpirational water loss rather than stomatal conductance alone. In beech, the highest WUE under EC × DS × N^+^ coincided with strong stimulation of *A*, whereas *G*s showed a more treatment-sensitive response, with a significant EC-induced reduction under DS × N^−^. In spruce, EC increased WUE particularly under N^+^ and DS, accompanied by higher *A* under N^+^, while *G*s was generally lower than in beech. Thus, beech showed a more plastic, resource-dependent coordination of carbon gain and stomatal regulation, whereas spruce maintained *A* at comparatively low stomatal conductance, consistent with a more conservative water-use strategy. These contrasting strategies are consistent with reports of stronger CO_2_-driven stomatal adjustment in broadleaved than coniferous species (Medlyn et al. 2001, Ofori-Amanfo et al. 2021, 2023, Poorter et al. 2022).

Because stomatal regulation during drought is closely coupled to ABA signalling (Waadt et al. 2022), the ABA responses provide additional insight into the contrasting water-use strategies of the two species. In spruce, drought under AC strongly increased ABA, particularly under N^−^, whereas this response was largely attenuated under EC and ABA remained comparatively stable across resource treatments. Because *G*s nevertheless remained low and WUE increased under EC, low stomatal conductance under EC was not associated with the strong ABA accumulation observed during drought under AC. In beech, ABA contents were generally much lower, and ABA declined significantly under EC × DS × N^−^ despite a simultaneous reduction in *G*s. Although EC-related declines in ABA have also been reported in other studies (Muhammad Aslam et al. 2022), our results indicate that, at the end of the drought-treatment period, ABA content did not closely track stomatal conductance in either species. This suggests species-specific coordination between hormonal status and stomatal regulation under EC, rather than a simple relationship between ABA accumulation and stomatal closure.

Overall, EC was the dominant driver of physiological and metabolic responses, while the magnitude of these responses was strongly modulated by water and nitrogen availability, supporting Hypothesis 1.

### Species-specific acclimation strategies under elevated CO_2_ and resource constraints

Previous studies have reported contrasting hydraulic and metabolic strategies in the more anisohydric beech and more isohydric spruce under environmental constraints (Košvancová et al. 2009, Pretzsch et al. 2014, Kolář et al. 2017, Ulrich and Grossiord 2023). Consistent with these differences, our data indicate a stronger resource-dependent plasticity of stomatal and metabolic responses in beech and a comparatively conservative response in spruce (Figures 2–4).

In beech, reduced stomatal conductance under EC does not necessarily translate into a proportional limitation of CO_2_ assimilation because internal CO_2_ transport also depends on mesophyll conductance and leaf anatomical traits (Flexas et al. 2012, Evans 2021). Together with the generally higher photosynthetic capacity of broadleaved than coniferous tree species (Košvancová et al. 2009, Leakey et al. 2009), this may explain why EC tends to stimulate *A* more strongly in beech, particularly when nitrogen was available (Figure 4). At the same time, EC reduced foliar nitrogen concentration and increased C:N ratios in both species (Figure S4 available as Supplementary Data at *Tree Physiology* Online), responses commonly reported under elevated CO_2_ (Simon et al. 2017, Ainsworth and Long 2021). In beech, the decline in foliar nitrogen coincided with reductions in *V*_Cmax_ and *J*_max_ across water and nitrogen treatments, consistent with photosynthetic downregulation. Nitrogen limitation can constrain investment in Rubisco and other photosynthetic proteins (Leakey et al. 2009, Körner 2022). In spruce, the stronger increase in C:N ratio was not accompanied by a decline in *V*_Cmax_ or *J*_max_ (Figure 5), indicating that lower foliar nitrogen did not translate into measurable biochemical downregulation in current-year needles. This apparent decoupling may reflect high nitrogen-use efficiency and the conservative nitrogen economy of spruce, which sustains photosynthetic function through efficient nitrogen reallocation within the photosynthetic apparatus (Warren et al. 2021). Additionally, it may be driven by age-dependent regulation of Rubisco activity and its characteristically high specific carboxylation activity (Košvancová et al. 2009, Urban et al. 2012, Lupi et al. 2013).

Metabolic responses further distinguished the two species. Consistent with a conservative strategy, spruce showed comparatively stable or only modest increases in soluble sugars under EC, and similarly stable TCA-cycle metabolites across water and nitrogen treatments; this was accompanied by a stronger investment in C-rich structural compounds and phenolic-based defence metabolism. Shikimic acid—a precursor for phenylpropanoids—was consistently higher in spruce than in beech across treatments (Figure 6), supporting the notion that conifers maintain a relatively high baseline investment in phenolics and lignin, even under resource-limited environments (Hammerbacher et al. 2020). Beech, in contrast, exhibited more plastic but also more stress-sensitive metabolic adjustment. Under EC and changing resource availability, *V*_Cmax_ and *J*_max_ declined, a pattern consistent with photosynthetic downregulation when drought and nitrogen limitation constrain nitrogen allocation to photosynthetic proteins and may increase the likelihood of sugar-mediated feedback regulation (Leakey et al. 2009, Körner 2022). Importantly, this decline in photosynthetic capacity co-occurred with coordinated shifts in primary and secondary metabolites (Figure 6, Figure S5 available as Supplementary Data at *Tree Physiology* Online), indicating stress accommodation rather than the maintenance of high carbon assimilation.

Divergent metabolite profiles further reinforce this contrast. Spruce maintained low and relatively stable SucA and glycine betaine (GB) contents across treatments, whereas beech showed sharp reductions under EC combined with drought and nitrogen limitation. In beech, lower GB under EC × DS may reflect (i) a reduced requirement for osmolyte accumulation when EC-induced stomatal closure improves plant water status (as indicated by Ψ; Figure S3 available as Supplementary Data at *Tree Physiology* Online), and/or (ii) a reallocation of carbon and nitrogen toward other protective or stress-related pathways (Chen and Murata 2011). Amino acid dynamics were similarly species-specific: beech reallocated nitrogen from several growth-related proteinogenic amino acids (e.g. phenylalanine, leucine, tryptophan) to stress-associated amino acids (e.g. proline, methionine, aspartic acid), which can support osmotic adjustment and antioxidative protection (Heinemann and Hildebrandt 2021). Spruce, in contrast, maintained a more stable amino acid profile, while preferentially accumulating phenolics (e.g. epicatechin, DL-catechin), which is consistent with an emphasis on antioxidant defence and long-term structural stability rather than short-term metabolic adjustment in response to acute stress conditions (Peñuelas et al. 1997, Hammerbacher et al. 2020, Sardans et al. 2020).

Together, these results support Hypothesis 2 by indicating contrasting, but not mutually exclusive, acclimation tendencies in the two species. Under the conditions of this experiment, beech adopted a flexible but stress-sensitive strategy involving a trade-off between metabolic plasticity and the maintenance of photosynthetic capacity under combined resource limitation. In contrast, spruce followed a more conservative strategy that sustained both photosynthetic capacity and high constitutive, phenolic-based defence with limited short-term plasticity. This divergence is also consistent with differences in leaf longevity: short-lived beech leaves may favour rapid metabolic adjustment over a single season, whereas long-lived spruce needles prioritize structural stability and sustained physiological performance.

### Metabolic feedbacks associated with species-specific photosynthetic regulation under elevated CO_2_

Building on the physiological patterns described particularly in Section ``Dominant effects of elevated CO_2_ and their modulation by resources'' and the contrasting acclimation strategies outlined in Section "Species-specific acclimation strategies under elevated CO_2_ and resource constraints", Hypothesis 3 proposed that regulation of photosynthetic capacity under EC is linked to species-specific metabolic adjustments. Our findings are broadly consistent with this hypothesis and suggest that *F. sylvatica* and *P. abies* adopt distinct physiological and biochemical strategies under EC that may contribute to differences in photosynthetic acclimation and stress resilience.

Beech consistently contained higher contents of soluble sugars (hexoses and disaccharides) than spruce, irrespective of CO_2_ treatment. This is consistent with reports that deciduous species often accumulate more soluble carbohydrates in leaves, whereas conifers rely more on starch reserves and maintain relatively stable sugar contents across environments (Schönbeck et al. 2018, Kaur et al. 2021). Although soluble sugars can contribute to osmotic adjustment (Kaur et al. 2021, Saddhe et al. 2021), elevated hexose contents have also been associated with feedback inhibition of photosynthesis when sink capacity is limited (Luo et al. 1999, Paul and Pellny 2003, Thompson et al. 2017). In this context, the comparatively lower hexose pool in spruce is consistent with a lower potential for sugar-mediated feedback regulation of photosynthetic capacity.

These carbohydrate-associated patterns were paralleled by differences in organic-acid profiles. In beech, high sugar contents under EC coincided with reduced SucA, particularly under EC × DS × N^−^. This pattern is consistent with a shift in carbon partitioning away from respiratory intermediates towards carbohydrate storage and/or secondary metabolism when carbon supply exceeds the capacity for growth under resource limitation (Dusenge et al. 2019). Notably, the decline in SucA was partially alleviated under N^+^, suggesting that nitrogen availability may modulate the balance between carbon utilization, growth demand and biosynthetic investments (Isah 2019). In spruce, by contrast, SucA remained consistently low across treatments. This does not necessarily imply low respiration; rather, it suggests that TCA-cycle intermediates were maintained within a comparatively narrow range, consistent with greater metabolic homeostasis across EC and resource treatments.

Shikimic acid further differentiated the investigated species. ShiA contents were approximately two orders of magnitude higher in spruce than in beech, which is consistent with a conifer strategy that may prioritize constitutive investment in phenolic-based defence and structural integrity via the shikimate–phenylpropanoid pathway (Witzell and Martín 2008, Hammerbacher et al. 2020). Such inherently high contents are compatible with a conservative metabolic strategy that maintains biosynthesis of lignin, phenolics and their precursors across diverse environments (Metsämuuronen and Sirén 2019, Qaderi et al. 2023). In our study, spruce showed only a moderate decline in ShiA under EC and maintained high contents regardless of water or nitrogen availability, consistent with comparatively stable phenolic defence metabolism across resource limitation. Beech, in contrast, showed a more variable response, with higher ShiA under EC × DS × N^+^ but lower ShiA under EC × WW × N^+^, consistent with greater resource sensitivity of shikimate metabolism and downstream phenylpropanoid pathway (Qaderi et al. 2023). Together, these contrasting patterns underline the distinct metabolic strategies: spruce prioritizes constitutive defence, whereas beech adjusts its phenolic metabolism more flexibly in response to resource availability.

Amino acid and phenolic profiles further supported these species-specific regulatory strategies. In beech, EC reduced several amino acids involved in protein synthesis and redox balance (e.g. phenylalanine, tyrosine, leucine, tryptophan, threonine), while several amino acids associated with stress responses (e.g. proline, methionine, and aspartic acid) showed higher contents under EC × DS × N^−^. This shift is consistent with a transition from growth-related processes towards protective functions, including osmotic adjustment and oxidative stress mitigation (Joshi et al. 2010, Hayat et al. 2012). Spruce, conversely, showed higher contents of leucine and phenylalanine under EC, which may reflect sustained amino-acid pools involved in stress signalling and phenylpropanoid metabolism (Jones and Jones 1997, Sardans et al. 2020). Elevated atmospheric CO_2_ also enhanced the accumulation of several phenolic compounds in spruce, including epicatechin and DL-catechin, especially under DS and N^−^, which may strengthen antioxidant and defensive capacity (Hasanuzzaman et al. 2020). Such coordinated adjustment of primary and secondary metabolism may have contributed to the ability of spruce to maintain photosynthetic capacity under EC while preserving a balance between growth and defence (Cui et al. 2024).

In summary, beech appears to rely on a more flexible but also more stress-sensitive metabolic adjustment, involving higher carbohydrate contents and stronger shifts in amino acids and organic acids, which was associated with reduced photosynthetic capacity under EC when water and nitrogen were limiting. Spruce, by contrast, appears to follow a more conservative metabolic strategy, characterized by comparatively stable photosynthetic capacity and constitutive investment in phenolic-based defence. Together, these patterns support Hypothesis 3 and suggest that species-specific metabolic adjustments and carbon–nutrient interactions underlie contrasting photosynthetic acclimation under CO_2_ enrichment.

## Conclusion

Elevated atmospheric CO_2_ was the strongest driver of physiological and metabolic variation in *F. sylvatica* and *P. abies*, while water and nitrogen availability strongly modified the magnitude of these responses, supporting Hypothesis 1. Although the EC level used here (~700 μmol mol^−1^) was higher than that commonly applied in field CO_2_-enrichment experiments, it provides a relevant test of tree responses to atmospheric CO_2_ concentrations that may occur later this century under high-emission scenarios. The imposed 5-week drought represented moderate water limitation, with a stronger physiological impact on beech than on spruce.

The species investigated adopted contrasting acclimation strategies under EC and resource limitation, supporting Hypothesis 2. Beech showed greater stomatal and metabolic plasticity, but this flexibility was accompanied by downregulation of photosynthetic capacity under EC. In contrast, spruce maintained comparatively stable photosynthetic capacity, generally low stomatal conductance, and a prominent phenolic-based defence metabolism, consistent with a more conservative physiological strategy.

Species-specific regulation of photosynthetic capacity was accompanied by contrasting metabolic adjustments, supporting Hypothesis 3. In beech, reduced photosynthetic capacity under EC coincided with resource-dependent shifts in organic-acid and amino-acid profiles associated with stress responses. In spruce, comparatively low sugar contents, stable TCA-cycle intermediates and high investment in phenylpropanoid-related metabolism were associated with the maintenance of photosynthetic capacity under EC despite resource constraints.

Together, these responses reveal two distinct acclimation strategies to elevated CO_2_ under declining water and nitrogen availability: beech relies on greater physiological and metabolic flexibility that may come at the cost of reduced photosynthetic capacity under prolonged resource limitation, whereas spruce maintains functional stability through a more conservative, defence-oriented metabolic strategy. Such species-specific differences may influence carbon uptake, competitive interactions, and forest dynamics under future climates characterized by rising CO_2_ and more frequent drought and nutrient limitations.

## Supplementary Material

SupplementaryFigures_Ofori_TP-2026-230_Revised_CLEAN_tpag125

## Data Availability

The data that support the findings of this study are openly available in the ASEP repository at https://doi.org/10.57680/asep.0652684.
