# Supplementary material for: Conservative versus flexible physiological and metabolic acclimation of Norway spruce and European beech under elevated CO2, nitrogen supply and water availability
Source: Tree Physiol. 2026 Sep 8;46(10):tpag125. doi: 10.1093/treephys/tpag125 (PMC13652638; doi:10.1093/treephys/tpag125)
Supplement: Supplementary file 1 [file supplementaryfigures_ofori_tp-2026-230_revised_clean_tpag125.docx]

**Supplementary Figures** to Kojo Kwakye Ofori-Amanfo et al.: Conservative versus flexible physiological and metabolic acclimation of Norway spruce and European beech under elevated CO_2_, nitrogen supply, and water availability (*Tree Physiology*)

**Supplementary Figure S1:** Seasonal courses of climatic drivers during the experimental period: photosynthetically active radiation (PAR) shown as daily sums, and relative air humidity (RH) and air temperature shown as daily means.

**Supplementary Figure S2**: Seasonal dynamics of soil volumetric water content in the ambient CO_2_ (A; AC) and elevated CO_2_ (B; EC) domes under well-watered (WW; blue) and drought-stressed (DS; red) treatments. AC, ambient CO_2_ concentration (~400 µmol CO_2_ mol^–1^); EC, elevated CO_2_ concentration (~700 µmol CO_2_ mol^–1^). The drought treatment started on 1 July (DOY 182); thereafter, WW plots received 20 mm irrigation per week, whereas irrigation was withheld from DS plots. Lines show measurements at 30-min intervals, with error bars indicating the standard error of the mean at weekly intervals.

**Supplementary Figure S3:** Boxplots showing midday leaf water potential (Ψ_noon_; upper panels) and UV-excited blue fluorescence (BF_UV; lower panels) in European beech (left) and Norway spruce (right) in response to CO_2_ treatment, water availability, and nitrogen supply. Measurements were performed at the end of the five-week water-limitation period in the second half of August. AC, ambient CO_2_ concentration (~400 µmol CO_2_ mol^–1^); EC, elevated CO_2_ concentration (~700 µmol CO_2_ mol^–1^); WW, well-watered (regular 20 mm irrigation per week during the drought-treatment period); DS, drought-stressed (no irrigation during the drought-treatment period); N^+^, nitrogen addition; N^–^, no nitrogen addition. Boxes show the 25th–75th percentiles, horizontal lines indicate medians, points indicate means, and whiskers extend to 1.5 × IQR. Different letters indicate significant differences among treatment combinations based on Tukey HSD tests (*p* ≤ 0.05). Asterisks indicate significant ANOVA main effects or interactions (* *p* < 0.05, ** *p* < 0.01, *** *p* < 0.001); “n.s.” indicates a non-significant effect.

**Supplementary Figure S4:** Boxplots showing foliar stoichiometry (C:N ratio and nitrogen content) in European beech (left) and Norway spruce (right) in response to CO_2_ treatment, water availability, and nitrogen supply. Measurements were performed at the end of the five-week water-limitation period in the second half of August. AC, ambient CO_2_ concentration (~400 µmol CO_2_ mol^–1^); EC, elevated CO_2_ concentration (~700 µmol CO_2_ mol^–1^); WW, well-watered (regular 20 mm irrigation per week during the drought-treatment period); DS, drought-stressed (no irrigation during the drought-treatment period); N^+^, nitrogen addition; N^–^, no nitrogen addition. Boxes show the 25th–75th percentiles, horizontal lines indicate medians, points indicate means, and whiskers extend to 1.5 × IQR. Different letters indicate significant differences among treatment combinations based on Tukey HSD tests (*p* ≤ 0.05). Asterisks indicate significant ANOVA main effects or interactions (* *p* < 0.05, ** *p* < 0.01, *** *p* < 0.001); “n.s.” indicates a non-significant effect.

**Supplementary Figure S5:** Boxplots showing effects of CO_2_ concentration (CO_2_), nitrogen supply (NS), and water availability (WA) on phenylalanine and leucine contents in beech (left) and spruce (right). Measurements were done at the end of the five-week water-limitation period in the second half of August. AC, ambient CO_2_ concentration (~400 µmol CO_2_ mol^–1^); EC, elevated CO_2_ concentration (~700 µmol CO_2_ mol^–1^); WW, well-watered (20 mm irrigation per week during the drought-treatment period); DS, drought-stressed (no irrigation during the drought-treatment period); N⁺, nitrogen addition; N⁻, no nitrogen addition. Boxes show the 25th–75th percentiles, horizontal lines indicate medians, points indicate means, and whiskers extend to 1.5 × IQR. Different letters indicate significant differences among treatment combinations based on Tukey HSD tests (*p* ≤ 0.05). Asterisks indicate significant ANOVA main effects or interactions (* *p* < 0.05, ** *p* < 0.01, *** *p* < 0.001); “n.s.” indicates a non-significant effect.

**Supplementary Figure S6:** Boxplots showing effects of CO_2_ concentration (CO_2_), nitrogen supply (NS), and water availability (WA) on hexoses and disaccharide contents in beech (left) and spruce (right). Measurements were done at the end of the five-week water-limitation period in the second half of August. See Supplementary Fig. S5 for plotting conventions, treatment abbreviations, and ANOVA notation. Different letters indicate significant differences among treatment combinations based on Tukey HSD tests (*p* ≤ 0.05).

**Supplementary Figure S7:** Boxplots showing effects of CO_2_ concentration (CO_2_), nitrogen supply (NS), and water availability (WA) on epicatechin and caffeic acid contents in beech (left) and spruce (right). Measurements were done at the end of the five-week water-limitation period in the second half of August. See Supplementary Fig. S5 for plotting conventions, treatment abbreviations, and ANOVA notation. Different letters indicate significant differences among treatment combinations based on Tukey HSD tests (*p* ≤ 0.05).

**Supplementary Figure S8:** Boxplots showing effects of CO_2_ concentration (CO_2_), nitrogen supply (NS), and water availability (WA) on abscisic acid (ABA) and glycine betaine (GB) contents in beech (left) and spruce (right). Measurements were done at the end of the five-week water-limitation period in the second half of August. See Supplementary Fig. S5 for plotting conventions, treatment abbreviations, and ANOVA notation. Different letters indicate significant differences among treatment combinations based on Tukey HSD tests (*p* ≤ 0.05).
